# Artificial Intelligence-Enabled Electrocardiogram Estimates Left Atrium Enlargement as a Predictor of Future Cardiovascular Disease

**DOI:** 10.3390/jpm12020315

**Published:** 2022-02-19

**Authors:** Yu-Sheng Lou, Chin-Sheng Lin, Wen-Hui Fang, Chia-Cheng Lee, Ching-Liang Ho, Chih-Hung Wang, Chin Lin

**Affiliations:** 1 Graduate Institutes of Life Sciences, National Defense Medical Center, No.161, Min-Chun E. Rd., Section 6, Neihu, Taipei 114, Taiwan; chaos53438@gmail.com; 2 School of Public Health, National Defense Medical Center, No.161, Min-Chun E. Rd., Section 6, Neihu, Taipei 114, Taiwan; 3 Division of Cardiology, Department of Internal Medicine, Tri-Service General Hospital, National Defense Medical Center, No 325, Cheng-Kung Rd., Section 2, Neihu, Taipei 114, Taiwan; littlelincs@gmail.com; 4 Department of Family and Community Medicine, Tri-Service General Hospital, National Defense Medical Center, No 325, Cheng-Kung Rd., Section 2, Neihu, Taipei 114, Taiwan; rumaf.fang@gmail.com; 5 Department of Medical Informatics, Tri-Service General Hospital, National Defense Medical Center, No 325, Cheng-Kung Rd., Section 2, Neihu, Taipei 114, Taiwan; lcgnet@gmail.com; 6 Division of Colorectal Surgery, Department of Surgery, Tri-Service General Hospital, National Defense Medical Center, No 325, Cheng-Kung Rd., Section 2, Neihu, Taipei 114, Taiwan; 7 Division of Hematology and Oncology, Tri-Service General Hospital, National Defense Medical Center, No 325, Cheng-Kung Rd., Section 2, Neihu, Taipei 114, Taiwan; charileho22623@gmail.com; 8 Department of Otolaryngology-Head and Neck Surgery, Tri-Service General Hospital, National Defense Medical Center, No 325, Cheng-Kung Rd., Section 2, Neihu, Taipei 114, Taiwan; chw@ms3.hinet.net; 9 Graduate Institute of Medical Sciences, National Defense Medical Center, No. 161, Min-Chun E. Rd., Section 6, Neihu, Taipei 114, Taiwan; 10 Medical Technology Education Center, School of Medicine, National Defense Medical Center, No. 161, Min-Chun E. Rd., Section 6, Neihu, Taipei 114, Taiwan

**Keywords:** artificial intelligence, electrocardiogram, deep learning, left atrium, left atrium enlargement, new-onset hypertension, new-onset stroke, new-onset mitral regurgitation, new-onset atrial fibrillation

## Abstract

Background: Left atrium enlargement (LAE) can be used as a predictor of future cardiovascular diseases, including hypertension (HTN) and atrial fibrillation (Afib). Typical electrocardiogram (ECG) changes have been reported in patients with LAE. This study developed a deep learning model (DLM)-enabled ECG system to identify patients with LAE. Method: Patients who had ECG records with corresponding echocardiography (ECHO) were included. There were 101,077 ECGs, 20,510 ECGs, 7611 ECGs, and 11,753 ECGs in the development, tuning, internal validation, and external validation sets, respectively. We evaluated the performance of a DLM-enabled ECG for diagnosing LAE and explored the prognostic value of ECG-LAE for new-onset HTN, new-onset stroke (STK), new-onset mitral regurgitation (MR), and new-onset Afib. Results: The DLM-enabled ECG achieved AUCs of 0.8127/0.8176 for diagnosing mild LAE, 0.8587/0.8688 for diagnosing moderate LAE, and 0.8899/0.8990 for diagnosing severe LAE in the internal/external validation sets. Notably, ECG-LAE had higher prognostic value compared to ECHO-LAE, which had C-indices of 0.711/0.714 compared to 0.695/0.692 for new-onset HTN, 0.676/0.688 compared to 0.663/0.677 for new-onset STK, 0.696/0.695 compared to 0.676/0.673 for new-onset MR, and 0.800/0.806 compared to 0.786/0.760 for new-onset Afib in internal/external validation sets, respectively. Conclusions: A DLM-enabled ECG could be considered as a LAE screening tool and provide better prognostic information for related cardiovascular diseases.

## 1. Introduction

Left atrium enlargement (LAE), identified by echocardiography (ECHO) with a prevalence of 16–16.5% in the general population [1,2], has been shown to be a reliable indicator for the risk of cardiovascular diseases (CVDs), such as hypertension (HTN) [3,4], stroke (STK) [5], mitral regurgitation (MR) [6,7], and atrial fibrillation (Afib) [8,9]. LAE might reflect the severity of diastolic dysfunction and abnormal left ventricle filling pressure [10]. Identifying LAE provides more information for preventing adverse events in many clinical scenarios. For example, transcatheter mitral valve repair had higher mortality and rehospitalization rates in patients with LAE [11]. Moreover, in patients with embolic STK of undetermined source and LAE, receiving anticoagulation therapy may reduce recurrent strokes [12]. Clinically, ECHO is commonly recommended to evaluate the left atrium (LA) size [13] due to its safety and accessibility compared to other LA imaging tools, such as cardiac magnetic resonance (CMR) and cardiac computed tomography [14].

An electrocardiogram (ECG) is a screening tool that easily captures cardiac structural signals [15] and is more accessible and inexpensive than ECHO. Previous studies have found that the P-wave ECG morphologies are associated with LAE patients, including a P wave greater than 100 msec, a P wave axis less than 30°, a notched P wave with an interpeak duration greater than 40 msec, and a P terminal force in lead V1 greater than 40 msec [16,17,18]. However, these ECG criteria have low accuracy for detecting LAE and may not be used in clinical practice [19,20]. With the evolution of deep learning models (DLMs), artificial intelligence (AI) with automatic ECG analysis exhibits outstanding capacities in detecting cardiovascular disorders, such as dyskalemias [21], myocardial infarction [22], digoxin toxicity [23], left ventricular systolic dysfunction [24], and even predicting mortalities [25]. Therefore, we hypothesized that a DLM-enhanced ECG interpretation may provide acceptable accuracy to identify LAE for large-scale community screening.

To our knowledge, there are few studies applying AI to detect LAE via an ECG. A previous study applied a DLM to segment an ECG and trained it with a traditional machine learning model for detecting LAE, which had a poor performance with an AUC of 0.62 [26]. Using DLM technology, a prior study showed an AUC of 0.95 for detecting LAE in a test dataset of 50 ECGs [27]. Although the superiority of DLMs has been demonstrated in LAE detection via an ECG, there has been no large-scale study to validate its performance in clinical practice. We conducted a retrospective multisite study at two hospitals to collect large-scale datasets. The baseline distributions of datasets were summarized in Appendix A. Moreover, a DLM has shown the ability to extract CVD previvors via an ECG [28], which could identify healthy patients with a risk of morbidities. Therefore, we aimed to apply an end-to-end DLM to an ECG for diagnosing LAE and evaluated the model performance in larger validation datasets. Finally, we investigated the prognostic value of a DLM-enabled ECG-LAE on LAE-related CVD outcomes.

## 2. Materials and Methods

### 2.1. Data Source and Population

This study was approved by the institutional review boards at Tri-Service General Hospital, Taipei, Taiwan (approval number: C202105049). Ethical review of this study was approved and patients’ informed consent was waived because data were in anonymized files and encrypted from the hospital to the data controller. Patient who visited from January 2011 to April 2021 at two separate institutions in the Tri-Service General Hospital system had their encrypted records stored in the data controller. We collected two datasets retrospectively from these two institutions. The first dataset was collected from an academic medical center (hospital A, NeiHu General Hospital), and the second dataset was collected from a community hospital (hospital B, Tingzhou Branch Hospital). These two hospitals were separately opened in 1999 and 1946; although, they belong to the same hospital group. We included patients who had at least one ECG and ECHO examination in this study.

**Figure 1:**
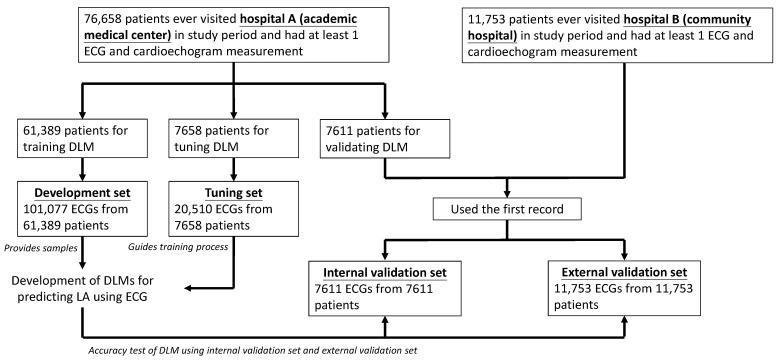
Development, tuning, internal validation, and external validation set generation and ECG labeling of the left atrium in a private dataset. Illustration of the dataset generation. This dataset creation was designed to assure the reliability and robustness of the data for training, tuning, and validation of the deep learning model. To avoid cross-contamination, once patients were included in one of the datasets, patients were not included in other datasets. The details of the workflow and the usage of each dataset are described in the Methods.

The generation of study datasets is shown in Figure 1. There were 76,658 patients with ECGs and corresponding labels in the study period from hospital A. To ensure the robustness of the DLM and maximize the follow-up period, we divided patients by the date of the first ECG examination. The earliest dataset was selected as the validation set, and the nearest dataset was selected as the development set. There were 61,389 patients with 101,077 ECG records in the development set, 7658 patients with 20,510 ECG records in the tuning set, and 7611 patients earlier than January 2016 in the internal validation set. To avoid the results being biased by patients having more ECGs, we only used the first ECG to validate the performance of the DLM for predicting left atrium, which was also used to follow-up for future diseases. Data from hospital B were only included in the external validation set. For the same reason, the first ECG records from patients in hospital B were used, and there were 11,753 ECGs from 11,753 patients. There was no overlap in patients among these sets.

### 2.2. Data Collection

ECGs involved the standard 12-lead ECG in this study and were collected using a Philips machine (PH080A, Philips Medical Systems, Andover, MA, USA). There were 5000 voltage–time trace signals for each lead (500 Hz sampling frequency for 10 s). We selected the nearest records of ECHO data within the 7 days before or after the ECG records as the corresponding ECG annotation. ECHO data were acquired using the Philips image system and were routinely measured by experienced cardiologists and technicians with a standardized method. We used the LA diameter of ECHO data to define LAE, and LAE was further classified as mild (>45 mm), moderate (>50 mm), and severe (>55 mm). The value of the LA diameter was limited from 20 mm to 65 mm to exclude outliers. We also compared the rule-based ECG analysis based on the Philips automatic system, which was parsed from the structured statements within the ECG reports. The diagnosis of LAE was confirmed with phrases within the reports, such as “Probable left atrial enlargement” and “Consider left atrial enlargement”. Patient characteristics, including sex, age, and body mass index, were collected from the electronic medical records in the system.

The complications during follow-up related to the primary outcome, LAE, were new-onset HTN, new-onset STK, new-onset MR, and new-onset Afib. Patients who conformed to the criteria during the follow-up period and did not meet any criteria before the date of an ECG examination were defined as having new-onset disease. Moderate or severe MR was defined as having MR in this study. The severity of MR was obtained from ECHO data, and was graded as minimal, mild, moderate, and severe. The definitions of other complications were based on the International Classification of Diseases, Ninth Revision and Tenth Revision (ICD-9 and ICD-10, respectively). HTN was defined as ICD-9 codes 401.x to 404.x and ICD-10 codes I10.x to I16.x, STK was defined as ICD-9 codes 430.x to 438.x and ICD-10 codes I60.x to I63.x, and Afib was defined as ICD-9 codes 427.31 and ICD-10 codes I48.x.

In addition to demographic and ECHO data, we also collected disease history, including diabetes mellitus (DM), hyperlipidemia (HLP), chronic kidney disease (CKD), coronary artery disease (CAD), HF, and chronic obstructive pulmonary disease (COPD). DM was confirmed by clinical criteria [29] or ICD-9 codes 250.x and ICD-10 codes E11.x. CKD was defined by a low estimated glomerular filtration rate and kidney damage [30] or ICD-9 codes 585.x and ICD-10 codes N18.x. HF was defined by an ejection fraction (EF) of ≤35% on ECHO data or ICD-9 codes 428.x, 398.91, and 402.x1 and ICD-10 codes I50.x in this study. The other disease histories were defined as follows: HLP (ICD-9: 272.x and ICD-10: E78.x), CAD (ICD-9: 410.x to 414.x, and 429.2 and ICD-10: I20.x to I25.x), and COPD (ICD-9: 490.x to 496.x and ICD-10: J44.9). Patients who were confirmed with the above criteria before the date of an ECG examination were identified as having a disease history.

### 2.3. Deep Learning Model for Estimating Left Atrium Diameter

We developed a DLM using 12-lead ECG trace signals as input to estimate the LA diameter. A DLM is a machine learning method that could automatically learn complex task from the raw data without hand-engineered features [31]. The DLM consists of many layers of artificial neurons and nonlinear transformation to produce the multiple levels of representation features. A DLM is suitable for image classification such as diseases and rhythms classification using raw ECG signals such as model inputs. We used the architecture of ECG12Net [21] as the base. ECG12Net, which has lead-specific blocks and an attention module, can effectively extract features from ECGs. The details of the DLM are described in Appendix B. We trained a DLM to predict the continuous values of the LA diameter using raw digital 12-lead ECG signals as input. The 12-lead ECG signals consisted of a 5000 × 12 matrix (5000 number sequences from each lead), and the 5000 sequences of each lead were randomly cropped into a length of 4096 as input during the training stage. To estimate the LA diameter, categorywise encoding technology and the training details were performed according to previous studies [22,23,32]. The details of categorywise encoding technology are described in Appendix C. The prediction output of the DLM is the continuous value of the LA diameter, and the prediction range of the LA diameter was limited from 20 to 65 mm.

### 2.4. Statistical Analysis and Model Performance Assessment

The model performance for predicting the LA diameter was tested in the internal and external validation sets. For continuous predictions, the mean difference (Diff), Pearson correlation coefficients (r), and mean absolute errors (MAE) were used. A receiver operating characteristic (ROC) curve was created to evaluate the performance for diagnosing mild, moderate, and severe LAE. Indicators of diagnostic accuracy included the area under the ROC curve (AUC), sensitivity, specificity, positive predictive value (PPV), and negative predictive value (NPV). We used the estimated the LA diameter from a DLM-enabled ECG to diagnose LAE. The best cutoff points of mild, moderate, and severe ECG-LAE were selected based on the highest Yunden’s index in the tuning set, which is the derivation of sensitivity, specificity, PPV and NPV.

The primary analysis was to assess the prognostic value of ECG-based LAE for new-onset CVDs. Stratified Kaplan–Meier (KM) analysis was performed to compare the prognostic ability of ECG-based LAE and ECHO-based LAE. The stratification was based on the severity of LAE, which classifies patients into without LAE, mild-to-moderate LAE, and severe LAE. Separate Cox proportional hazards models were also fit using either ECG-based LAE or ECHO-based LAE as predictor variables, and hazard ratios (HRs) and C-indices were used to compare the prognostic performances. A stratified analysis was further conducted to explore the potential influence of disease history on predicting new-onset complications. All Cox models were adjusted for sex and age, and risk analyses were performed on the follow-up data in the internal and external validation sets. All statistical analyses were carried out using the R language (version 3.4.4).

## 3. Results

Table 1 summarizes the baseline characteristics of patients and their follow-up data for new-onset diseases. This study included patients with a mean (standard deviation, SD) age of 63.9 (17.4), 68.1 (16.3), 63.5 (16.6), and 65.8 (18.1) years in the development, tuning, internal, and external validation sets, respectively. There were 989/1186, 592/693, 687/815, and 494/745 patients who developed new-onset HTN, STK, MR, and Afib over median (interquartile range, IQR) follow-up years of 2.0 (0.3–4.4)/1.2 (0.2–3.2), 3.2 (1.0–5.4)/2.2 (0.6–4.4), 2.8 (1.3–4.8)/2.6 (1.1–4.4), and 3.2 (1.0–5.5)/2.3 (0.6–4.5) in the internal/external validation sets, respectively.

**Figure 2:**
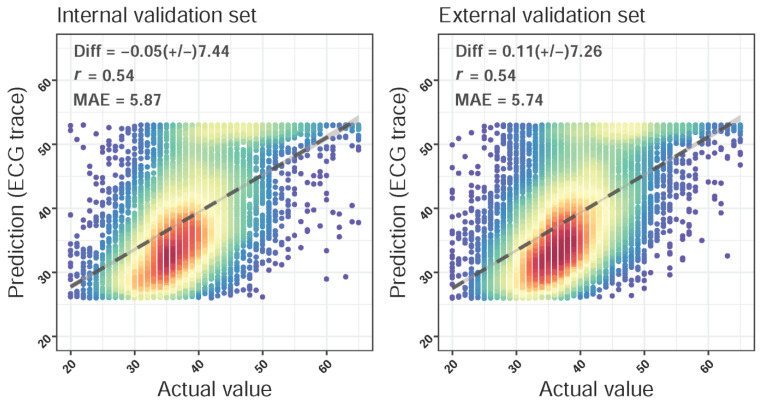
Scatter plots of the predicted left atrium (ECG-LA) diameter via an ECG only compared to the actual left atrium (LA) diameter. The *x*-axis indicates the actual LA diameter, and the *y*-axis presents the ECG-LA diameter. The highest density is represented by red points, followed by yellow, green, light blue, and dark blue points. We presented the mean difference (Diff), Pearson correlation coefficients (COR), and mean absolute errors (MAE) to demonstrate the accuracy of the DLM. The black lines with 95% confidence intervals are fitted via simple linear regression.

We tested the performance of the DLM for the LA diameter estimation. Scatter plots with the actual value of the LA diameter versus ECG-LA diameter are presented in Figure 2. In the internal/external validation sets, the mean difference (SD) was −0.05 (7.44)/0.11 (7.26) mm, the Pearson correlation coefficients were 0.54/0.54, and the MAE was 5.87/5.74 mm. The results demonstrated a general slight underestimation of the LA diameter by the DLM.

**Figure 3:**
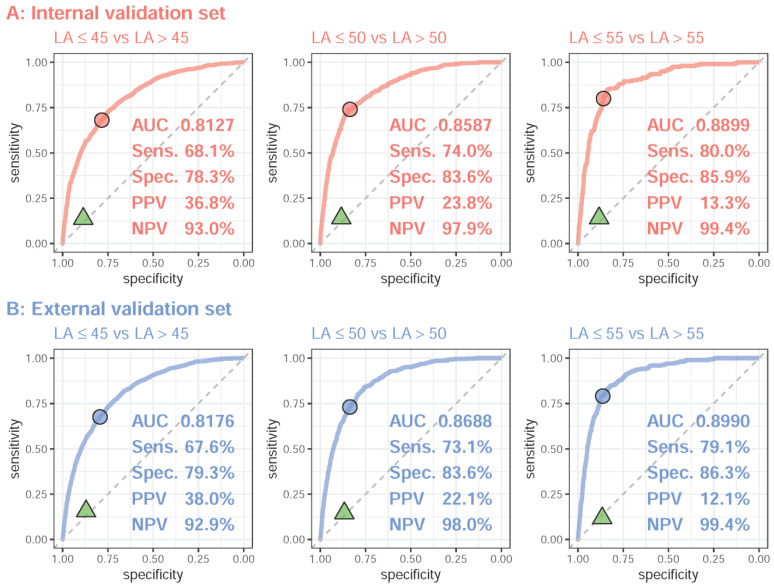
Receiver operating characteristic (ROC) curve analysis for mild to severe left atrium enlargement (LAE) from deep learning model-based ECG voltage–time traces. The ROC curve (*x*-axis = specificity and *y*-axis = sensitivity) and the area under the ROC curve (AUC) were calculated using the internal validation set and external validation set. The triangles denote the performance of the LAE diagnosis from the rule-based ECG analysis. The operating point was selected based on the maximum Yunden’s index in the tuning set, which was used to calculate the corresponding sensitivities and specificities in the two validation sets.

We next evaluated the DLM performance for diagnosing LAE using the ECG-LA diameter. Figure 3 shows the ROC curves for mild to severe LAE from the DLM. For the classification of mild LAE, moderate LAE, and severe LAE, the AUCs were 0.8127/0.8176, 0.8587/0.8688, and 0.8899/0.8990 with a sensitivity of 68.1%/67.6%, 74.0%/73.1%, and 80.0%/79.1% and specificity of 78.3%/79.3%, 83.6%/83.6%, and 85.9%/86.3% in the internal/external validation sets, respectively. The identical AUCs in the validation sets demonstrated the robustness of ECG-LAE to different datasets. We further compared the performance of the DLM and rule-based ECG analysis based on the Philips automatic system. Using the diagnosis from the rule-based ECG analysis, the sensitivities for mild, moderate, and severe LAE were 0.137/0.156, 0.140/0.145, and 0.140/0.117, and the specificities were 0.886/0.870, 0.884/0.867, and 0.883/0.866 in the internal/external validation sets, respectively. These results demonstrated significantly lower sensitivities of the ECG measure compared to those of the DLM. Notably, the DLM had high NPVs of 93.0%/92.9%, 97.9%/98.0%, and 99.4%/99.4% with relatively low PPVs of 36.8%/38.0%, 23.8%/22.1%, and 13.3%/12.1% for mild, moderate, and severe LAE in internal/external validation sets, respectively. Although low PPVs of the DLM were related to prevalence, the high NPVs suggest that ECG-LAE could be used as a screening tool in clinical practice.

**Figure 4:**
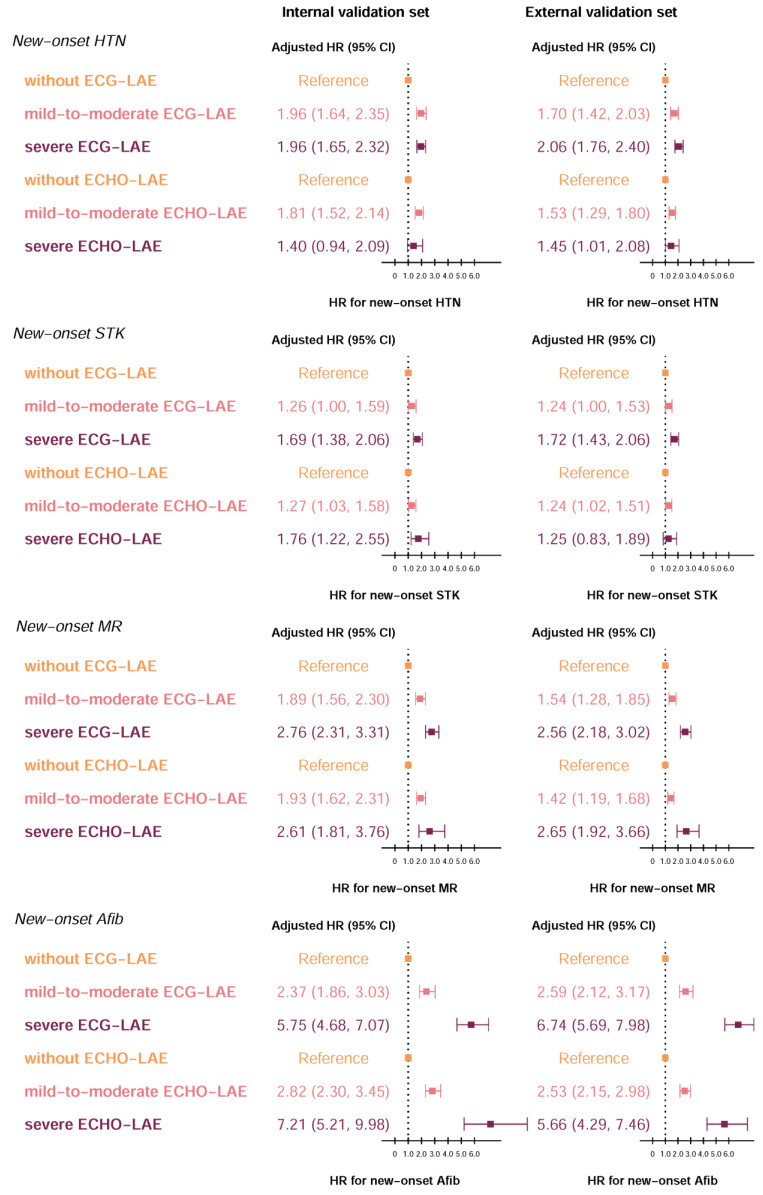
Forest plots of the adjusted hazard ratio for each severity of electrocardiogram-based left atrium enlargement (ECG-LAE) and echocardiography-based left atrium enlargement (ECHO-LAE) on new-onset complications. The cutoff points of without, mild-to-moderate, and severe LAE were defined as 45 and 55 mm, respectively. The analyses were conducted in both internal and external validation sets. Hazard ratios are adjusted for sex and age. Abbreviations: HR, Hazard ratios; CI, confidence interval.

ECHO-LAE may be an early sign of new-onset HTN [3,4], STK [5], MR [6,7], and Afib [8,9]. Although the ECG-LA diameter might be inconsistent with the ECHO-LA diameter, we explored the prognostic value of ECG-LAE in new-onset HTN, STK, MR, and Afib. The KM curves for patients with severe, mild-to-moderate, or without LAE grouped by ECHO-LAE and ECG-LAE were summarized in Appendix D. A total of 3582/5087 at-risk patients were included in the new-onset HTN analysis, 6247/9347 at-risk patients were included in the new-onset STK analysis, 3342/4492 at-risk patients were included in the new-onset MR analysis, and 7033/10,763 at-risk patients were included in the new-onset Afib analysis in the internal/external validation sets. In internal validation sets, among patients identified by the DLM as having a mid-to-moderate/severe ECG-LAE, the cumulative incidence rates for new-onset HTN, STK, MR, and Afib were 44.7%/49.8%, 9.6%/13.7%, 13.5%/25.6%, and 8.1%/20.2% at 2 years and 59.0%/64.1%, 15.8%/24.4%, 45.2%/55.1%, and 16.7%/34.0% at 6 years, respectively. In the external validation set, a similar trend of higher long-term risks in ECG-LAE patients was also observed. As expected, the cumulative incidence curves were obviously different in each severity group by ECG-LAE. Importantly, ECG-LAE demonstrated its superior ability to discriminate between severe and mild-to-moderate populations for the development of new-onset HTN in both validation sets. We further investigated the prognostic performance using age and sex adjustments. Of note, the superiorities of ECG-LAE with gender- and age-adjusted were evident compared to ECHO-LAE, which showed higher C-indices of 0.711/0.714 compared to 0.695/0.692 in new-onset HTN analysis, 0.676/0.688 compared to 0.663/0.677 in new-onset STK analysis, 0.696/0.695 compared to 0.676/0.673 in new-onset MR analysis, and 0.800/0.806 compared to 0.786/0.760 in new-onset Afib analysis in internal/external validation sets. Figure 4 shows the forest plots of the adjusted hazard ratio for patients with severe, mild-to-moderate, or without LAE grouped by ECHO-LAE and ECG-LAE. In the internal validation set, the adjusted HRs (95% confidence interval) of developing new-onset HTN, STK, MR, and Afib were 1.96 (1.65 to 2.32), 1.69 (1.38 to 2.06), 2.76 (2.31 to 3.31), and 5.75 (4.68 to 7.07) for severe ECG-LAE, respectively, compared with patients identified as without LAE. For mild-to-moderate ECG-LAE, the adjusted HRs of new-onset HTN, STK, MR, and Afib were 1.96 (1.64 to 2.35), 1.26 (1.00 to 1.59), 1.89 (1.56 to 2.30), and 2.37 (1.86 to 3.03), respectively. Similar results of the adjusted HRs by ECG-LAE were also observed in the external validation set. These results demonstrated that ECG-LAE had a better prognostic ability for future CVDs than ECHO-LAE.

**Figure 5:**
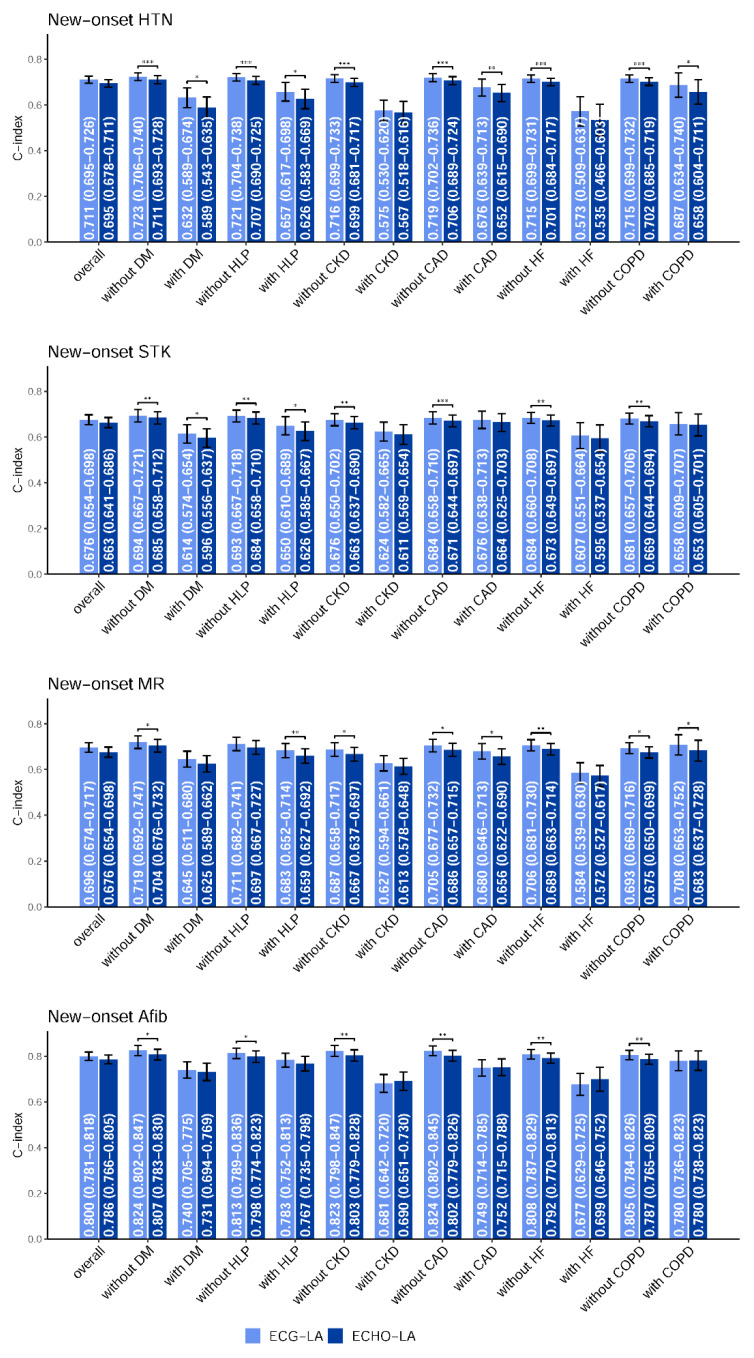
Stratified analysis for the C-index comparison between electrocardiogram-based left atrium (ECG-LA) diameter and echocardiography-based left atrium (ECHO-LA) diameter on new-onset complications in internal validation set. The analyses were stratified by the disease histories of the populations. The C-index was calculated based on the ECG-LA/ECHO-LA combined with sex and age. *: *p* < 0.05; **: *p* < 0.01; ***: *p* < 0.001. The overall population analyses were performed with an unstratified Cox proportional-hazards model.

Since the risk of CVDs might be associated with a personal history of diseases, we further conducted stratified analyses to evaluate the prognostic performance for new-onset CVDs among patients with and without a disease history. Figure 5 summarizes the C-index of the separate Cox model using ECG-LAE and ECHO-LAE as predictors in the internal validation set, and the analysis results for the external validation set were shown in Appendix E. Cox models were all adjusted with gender and age. Compared to patients without history, ECG-LAE and ECHO-LAE had lower prognostic ability with lower C-indices in patients with disease history. In the internal validation set, ECG-LAE achieved significantly higher C-indices than ECHO-LAE for new-onset HTN (C-index: 0.723 to 0.711, *p* < 0.001), STK (C-index: 0.694 to 0.685, *p* < 0.01), MR (C-index: 0.719 to 0.704, *p* < 0.05), and Afib (C-index: 0.824 to 0.807, *p* < 0.05) in patients without a history of DM. Similar trends of higher C-indices provided by ECG-LAE were also observed regardless of whether the patient had a history of HLP, CAD, or COPD. The reason for the different results in patients with CKD history and with HF history might be due to the small number of those patients in each new-onset disease analysis. These results highlighted the strength of ECG-LAE, which could provide more information on future CVDs, especially in patients without disease histories.

## 4. Discussion

In this study, we applied the DLM with a development set of more than 50,000 ECGs for diagnosing LAE. A DLM-enabled ECG provided better performances with AUCs of 0.813/0.818, 0.859/0.869, and 0.890/0.899 on mild, moderate, and severe LAE detection in the internal/external validation sets, respectively, than that of the automatic analysis system. In addition to accurately diagnosing LAE, ECG-LAE had a better prognostic role than ECHO-LAE for new-onset HTN, STK, MR, and Afib, which are known to be associated with a history of LAE [5,8,9,33]. We proposed that ECG-LAE provides more information on new-onset CVDs than ECHO-LAE, regardless of whether patients have a history of CVD-related diseases.

ECHO is a commonly used and noninvasive method that provides thin cross-sections of cardiac structures and cardiac anatomy, such as the left and right atrium, left and right ventricles, and valvular structures [34]. ECHO-LA can be used to predict cardiovascular events [35] and serve as a prognostic marker for CVD [10]. In our study, we found that the application of the DLM to the standard 12-lead ECG enabled accurate detection of LAE with AUCs of 0.81 to 0.90 in different severities. Previous rule-based criteria based on the ECG intervals and magnitudes only exhibited AUCs less than 0.60 for diagnosing LAE [20], which was consistent with our Philips automatic system. The DLM has demonstrated its outstanding performance in ECG analysis, which has shown better accuracy than physicians in diagnosing dyskalemia [21], detecting acute myocardial infarction [22], and detecting arrhythmia [36]. Likewise, our ECG-LAE with high NPVs gave us the opportunity to exclude patients in clinical practice. Although a previous study suggested a DLM to diagnose LAE with an AUC of 0.95 in a test dataset of 50 ECGs [27], our larger validation datasets provide a more realistic result in the real world. Moreover, we demonstrated the continuous predictions of the LA diameter with MAEs of 5.87/5.74 in the internal/external validation set based on our larger development set. Therefore, a DLM has the potential to screen the high-risk population of LAE via an ECG, thereby improving the quality of care for such patients.

LAE has been found to be a predictor of new-onset HTN [3,4], STK [5], MR [6,7], and Afib [8,9]. HTN is the leading risk factor for CVD, and is often overlooked due to the largely asymptomatic population [37]. Additionally, the risk assessment of STK is crucial for further STK prevention [38,39]. Furthermore, valvular heart disease with MR is often underdiagnosed and needs to be closely monitored in the community [40]. Afib, the critical factor of CVD, is often asymptomatic [41], but is strongly associated with an increased risk of STK [42]. In addition to screening LAE, our study shows that a DLM-enabled ECG system for diagnosing LAE could provide more information on future CVDs. Previous studies have shown that a DLM has the ability to predict disease previvors via an ECG [28]. For example, a DLM-enabled ECG could identify patients who had a risk of developing a low ejection fraction (EF) but had an initially normal EF [24]. Moreover, the deviation between the DLM-predicted age via an ECG and the chronologic age might be associated with CVDs [43], which could be used as a predictor of mortality [44]. Of note, previous studies have found that the ECG criteria of P-wave morphologies are associated with LAE [16,17]. One of the ECG criteria for LAE has been reported to identify patients with more disabling STK [45]. P-wave terminal force in lead V1 is related to LA abnormalities associated with STK independent of Afib [46]. P-wave abnormalities have also been previously linked to Afib and pre-HTN [47,48]. Interestingly, ECG-LAE and ECHO-LAE have the lower prognostic ability for new-onset CVDs among patients with disease histories in the stratified analysis. This results might imply that those co-morbidities are potential confounding factors for new-onset CVDs. In other words, ECG-LAE and ECHO-LAE could provide more prognostic information in these patients without co-morbidities, and ECG-LAE has shown its superior ability to predict new-onset CVDs compared to ECHO-LAE in this study. Based on these relationships among LAE, the related diseases, and the ECG morphologies, our DLM-enabled ECG for LAE, therefore, had the potential to learn information about the disease previvors of new-onset HTN, STK, MR, and Afib.

An ECG has the advantage of low costs compared to performing ECHO. A previous randomized controlled trial (RCT) demonstrated that a DLM-enabled ECG could identify undiscovered low-EF patients confirmed by ECHO, and a DLM-enabled ECG intervention increased the diagnosis of low EF [49]. Screening for new-onset HTN, STK, MR, and Afib would provide an opportunity to prevent certain CVD outcomes and reduce mortality. It has been suggested that the risk reductions in STK and death are similar in patients with screen-detected Afib and in those with incidentally detected Afib [50]. In a community-based RCT study, there were fewer annual hospital admissions for CVD with the intervention of blood pressure screenings [51]. As mentioned above, early-detected MR with prompt management helps to improve the clinical outcome [52]. Therefore, our DLM-enabled ECG system could be used as a convenient and low-cost screening tool with superior ability to identify patients with LAE and provide information to reduce their risk of further cardiovascular events.

There were some limitations presented in this study. First, the patients in this study were retrospectively enrolled from hospitals. A prospective study should be considered to validate the application and value of ECG-LAE in a community-based population. Second, left atrium volume (LAV) has been described to have a higher association with cardiovascular disease than the LA diameter [1]. In this study, we used the predictive LA diameter to diagnose LAE because the LA diameter was easier to acquire in most patients. Further studies of DLMs using LAV to identify LAE could be explored. Third, there were a small number of patients with CKD history and HF history who developed new-onset HTN, STK, MR, and Afib in our dataset. ECG-LAE should be applied to a larger population of patients with a history of those diseases to explore the capacity of a DLM-enabled ECG. Finally, a limitation that cannot be ignored for a DLM is its explainability. Although the rule-based ECG criteria and traditional statistical methods could provide a more comprehensive interpretation between the ECG morphology and physiology, the performance of the DLM was superior to that of traditional methods. Additional studies are necessary to explore the association between ECG morphology findings and LAE, especially their relationship to new-onset HTN, STK, MR, and Afib.

## 5. Conclusions

In this study, we applied a DLM to detect LAE by an ECG, and enabled an ECG as an accurate screening tool for diagnosing LAE and for predicting the severity of LAE. Our study demonstrated that a DLM-enabled ECG for diagnosing LAE could provide additional prognostic information on new-onset HTN, STK, MR, and Afib. Although further research is needed, our DLM-enabled ECG system gives us the opportunity to promote health care in patients with asymptomatic LAE.

## Figures and Tables

**Table 1:** Baseline characteristics.

	Development Set	Tuning Set	Internal Validation Set	External Validation Set	*p*-Value
Demography					
Sex (male)	51,834 (53.8%)	10,812 (52.7%)	3854 (50.6%)	5834 (49.6%)	<0.001
Age (years)	63.9 ± 17.4	68.1 ± 16.3	63.5 ± 16.6	65.8 ± 18.1	<0.001
BMI (kg/m^2^)	24.6 ± 4.4	24.3 ± 4.4	24.5 ± 4.3	24.4 ± 4.3	<0.001
Disease history					
DM	22,877 (23.7%)	7351 (35.8%)	2261 (29.7%)	3651 (31.1%)	<0.001
HLP	28,925 (30.0%)	9206 (44.9%)	3142 (41.3%)	5197 (44.2%)	<0.001
CKD	23,284 (24.2%)	8987 (43.8%)	1861 (24.5%)	2911 (24.8%)	<0.001
CAD	26,774 (27.8%)	8394 (40.9%)	2362 (31.0%)	3652 (31.1%)	<0.001
HF	12,701 (13.2%)	4852 (23.7%)	953 (12.5%)	1492 (12.7%)	<0.001
COPD	12,138 (12.6%)	4464 (21.8%)	1505 (19.8%)	2778 (23.6%)	<0.001
Echocardiography data					
LA (mm)	38.4 ± 7.4	39.5 ± 7.9	38.5 ± 7.5	38.7 ± 7.2	<0.001
LV-D (mm)	47.5 ± 7.1	47.9 ± 7.8	47.3 ± 7.1	47.1 ± 6.8	<0.001
LV-S (mm)	30.3 ± 6.9	31.2 ± 7.8	29.8 ± 6.8	29.6 ± 6.3	<0.001
IVS (mm)	11.2 ± 2.6	11.5 ± 2.6	11.2 ± 2.6	11.1 ± 2.6	<0.001
LVPW (mm)	9.3 ± 1.7	9.5 ± 1.8	9.3 ± 1.7	9.1 ± 1.7	<0.001
AO (mm)	32.7 ± 4.4	33.1 ± 4.4	32.8 ± 4.5	32.8 ± 4.3	<0.001
RV (mm)	23.8 ± 5.0	24.2 ± 5.1	24.1 ± 5.1	24.0 ± 4.9	<0.001
PASP (mmHg)	33.3 ± 11.2	34.7 ± 12.4	32.1 ± 10.3	32.9 ± 10.7	<0.001
PE (mm)	0.5 ± 2.1	0.6 ± 2.1	0.3 ± 1.8	0.4 ± 1.7	<0.001
EF (%)	63.5 ± 12.6	61.0 ± 14.3	65.2 ± 11.4	65.4 ± 10.8	<0.001
Follow up data					
Present HTN		11,951 (58.3%)	3971 (52.2%)	6500 (55.3%)	<0.001
Follow-up (years), median (IQR)		0.9 (0.1–2.8)	2.0 (0.3–4.4)	1.2 (0.2–3.2)	
New-onset HTN		2708 (32.4%)	989 (27.6%)	1186 (23.3%)	
Present STK		4661 (22.7%)	1286 (16.9%)	2189 (18.6%)	<0.001
Follow-up (years), median (IQR)		2.0 (0.5–3.3)	3.2 (1.0–5.4)	2.2 (0.6–4.4)	
New-onset STK		1274 (8.2%)	592 (9.5%)	693 (7.4%)	
Present MR		3677 (17.9%)	835 (10.9%)	1324 (11.3%)	<0.001
Follow-up (years), median (IQR)		1.8 (0.8–3.1)	2.8 (1.3–4.8)	2.6 (1.1–4.4)	
New-onset MR		1976 (22.8%)	687 (20.6%)	815 (18.1%)	
Present Afib		2622 (12.8%)	496 (6.5%)	756 (6.4%)	<0.001
Follow-up (years), median (IQR)		1.8 (0.4–3.3)	3.2 (1.0–5.5)	2.3 (0.6–4.5)	
New-onset Afib		1670 (9.5%)	494 (7.0%)	745 (6.9%)	

Abbreviations: BMI, body mass index; DM, diabetes mellitus; HLP, hyperlipidemia; CKD, chronic kidney disease; CAD, coronary artery disease; HF, heart failure; COPD, chronic obstructive pulmonary disease; LA, left atrium; LV-D, left ventricle (end-diastole); LV-S, left ventricle (end-systole); IVS, interventricular septum; LVPW, left ventricular posterior wall; AO, aortic root; RV, right ventricle; PASP, pulmonary artery systolic pressure; PE, pericardial effusion; EF, ejection fraction; HTN, hypertension; STK, stroke; MR, mitral regurgitation; Afib, atrial fibrillation.

**Figure A1:**
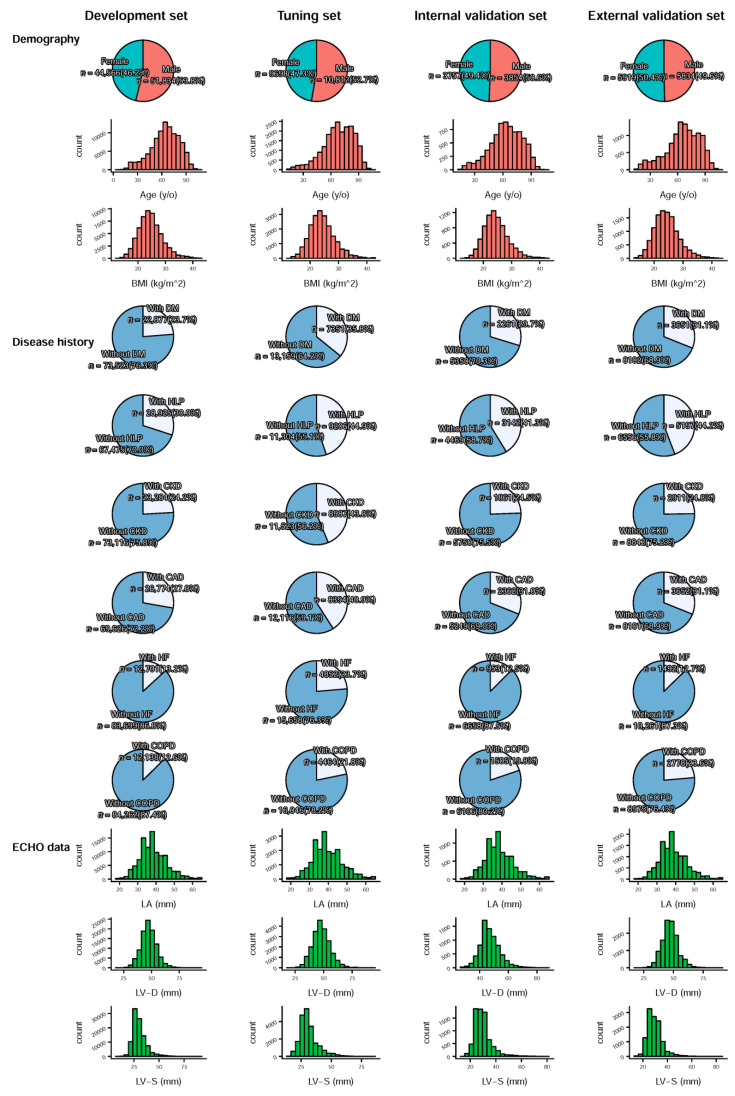

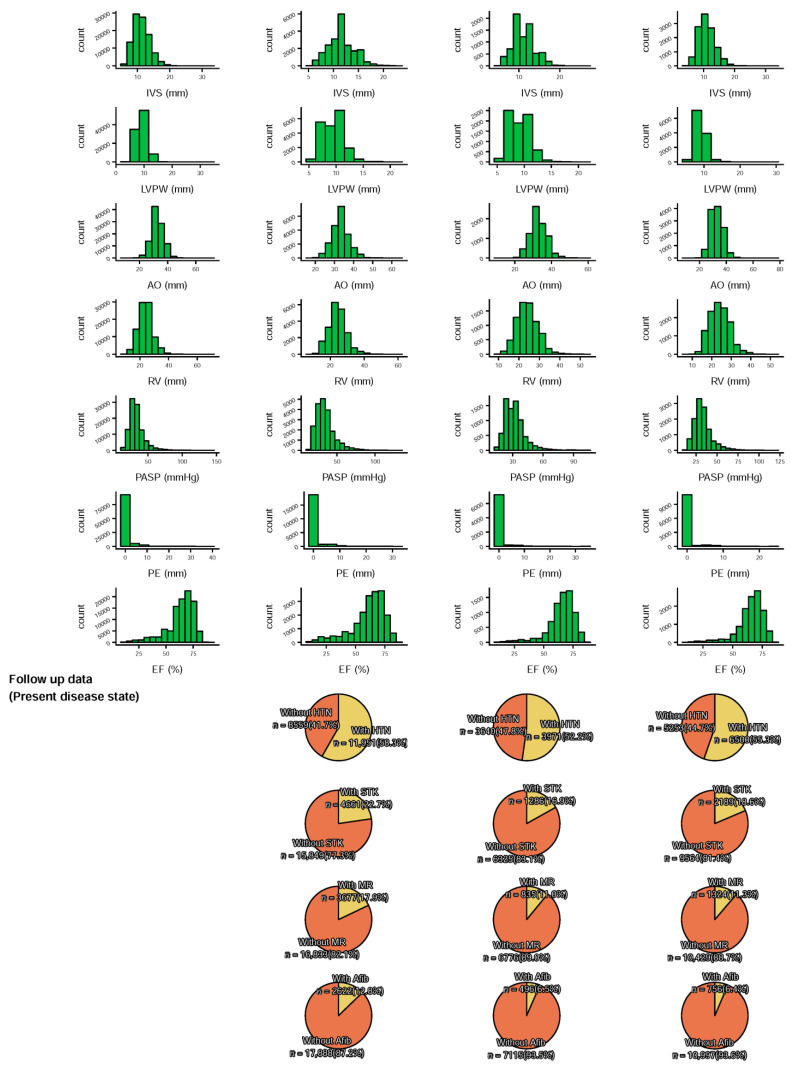
Summary of the baseline distributions in development, tuning, internal validation, and external validation sets. Abbreviations: ECHO, echocardiography; BMI, body mass index; DM, diabetes mellitus; HLP, hyperlipidemia; CKD, chronic kidney disease; CAD, coronary artery disease; HF, heart failure; COPD, chronic obstructive pulmonary disease; LA, left atrium; LV-D, left ventricle (end-diastole); LV-S, left ventricle (end-systole); IVS, interventricular septum; LVPW, left ventricular posterior wall; AO, aortic root; RV, right ventricle; PASP, pulmonary artery systolic pressure; PE, pericardial effusion; EF, ejection fraction; HTN, hypertension; STK, stroke; MR, mitral regurgitation; Afib, atrial fibrillation.

**Figure A2:**
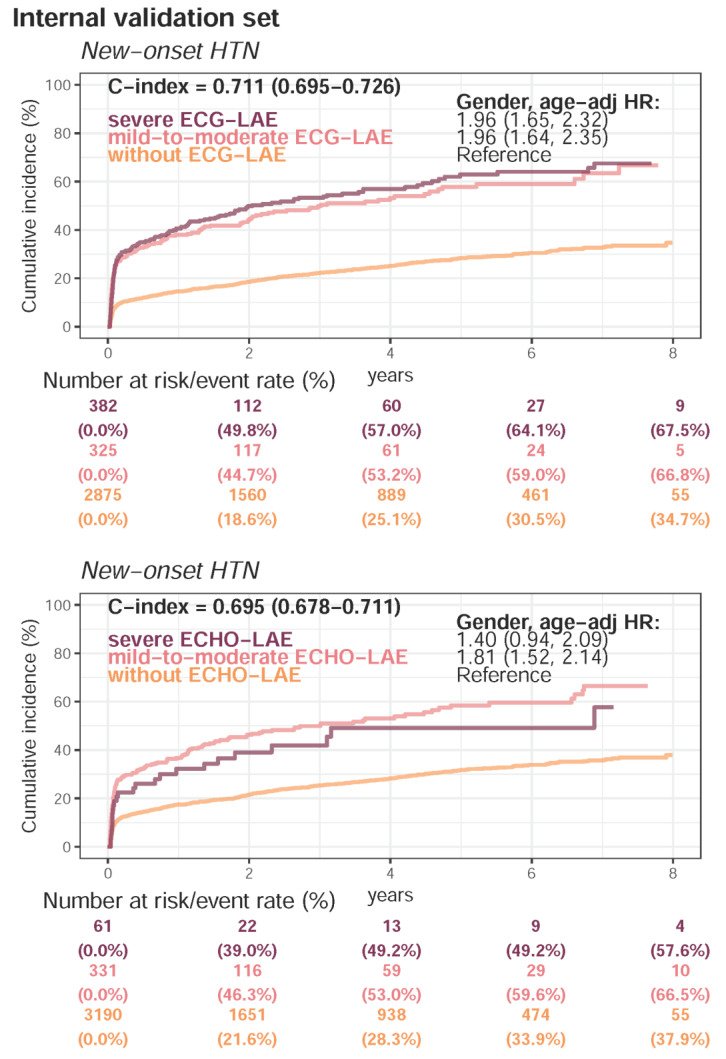
Kaplan–Meier curves for each severity of electrocardiogram-based left atrium enlargement (ECG-LAE) and echocardiography-based left atrium enlargement (ECHO-LAE) on new-onset hypertension (HTN) in internal validation set. The cutoff points of without, mild-to-moderate, and severe LAE were defined as 45 and 55 mm, respectively. The C-index was calculated based on the continuous value combined with sex and age. The table shows the at-risk population and cumulative risk for the given time intervals in each risk stratification.

**Figure A3:**
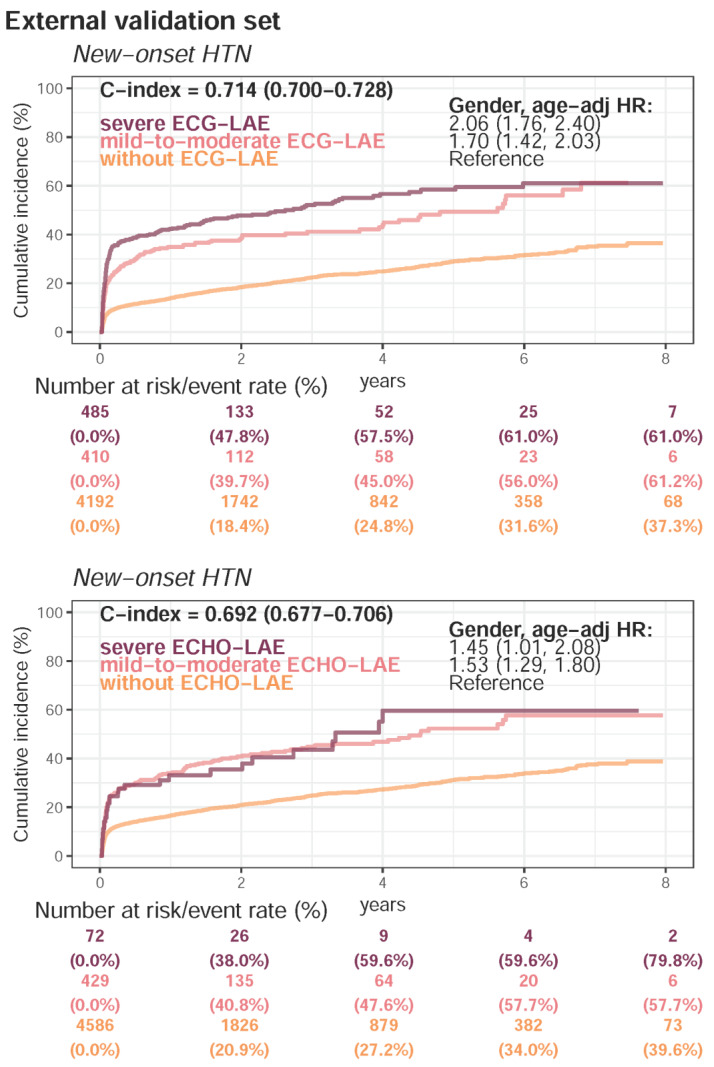
Kaplan–Meier curves for each severity of electrocardiogram-based left atrium enlargement (ECG-LAE) and echocardiography-based left atrium enlargement (ECHO-LAE) on new-onset hypertension (HTN) in external validation set. The cutoff points of without, mild-to-moderate, and severe LAE were defined as 45 and 55 mm, respectively. The C-index was calculated based on the continuous value combined with sex and age. The table shows the at-risk population and cumulative risk for the given time intervals in each risk stratification.

**Figure A4:**
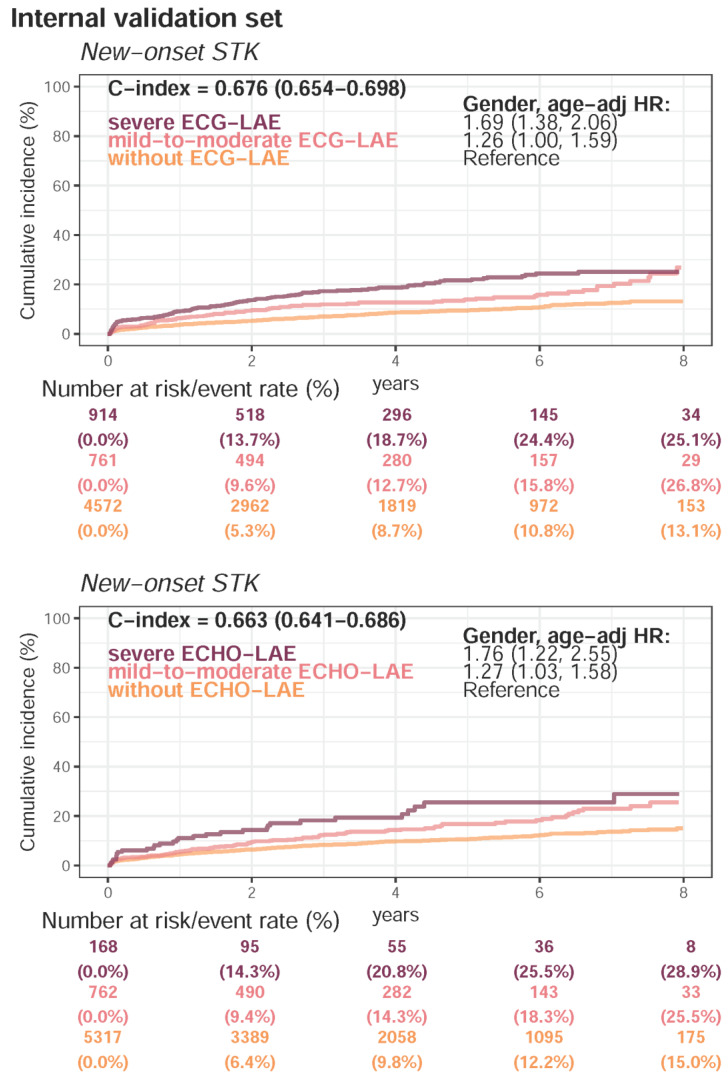
Kaplan–Meier curves for each severity of electrocardiogram-based left atrium enlargement (ECG-LAE) and echocardiography-based left atrium enlargement (ECHO-LAE) on new-onset stroke (STK) in internal validation set. The cutoff points of without, mild-to-moderate, and severe LAE were defined as 45 and 55 mm, respectively. The C-index was calculated based on the continuous value combined with sex and age. The table shows the at-risk population and cumulative risk for the given time intervals in each risk stratification.

**Figure A5:**
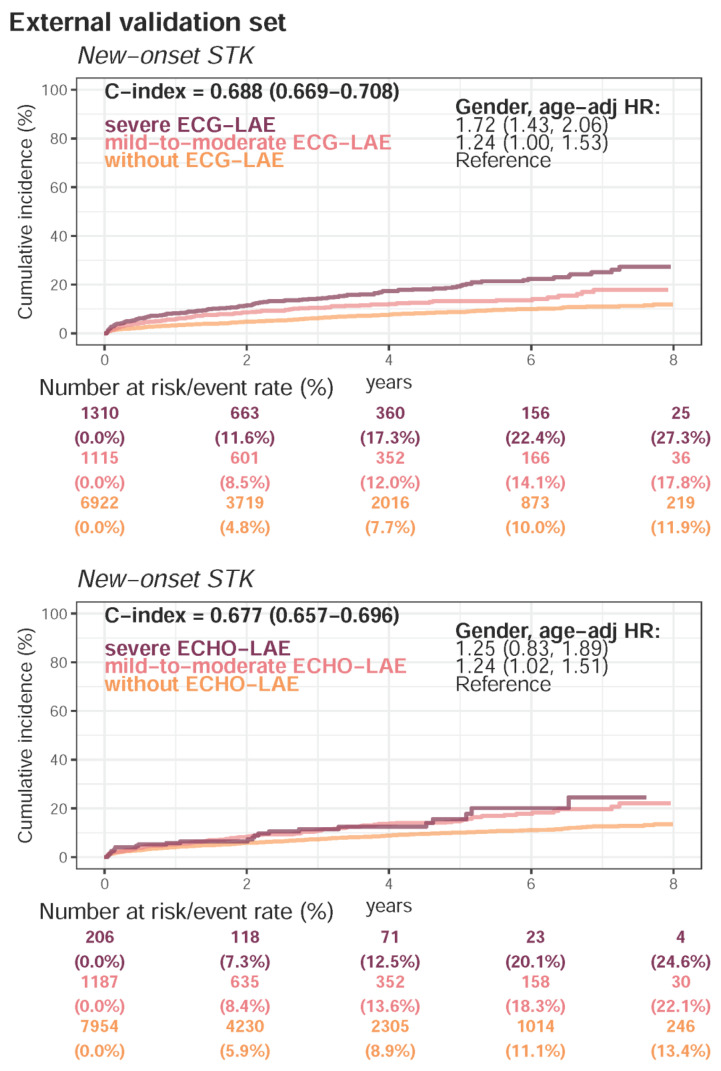
Kaplan–Meier curves for each severity of electrocardiogram-based left atrium enlargement (ECG-LAE) and echocardiography-based left atrium enlargement (ECHO-LAE) on new-onset stroke (STK) in external validation set. The cutoff points of without, mild-to-moderate, and severe LAE were defined as 45 and 55 mm, respectively. The C-index was calculated based on the continuous value combined with sex and age. The table shows the at-risk population and cumulative risk for the given time intervals in each risk stratification.

**Figure A6:**
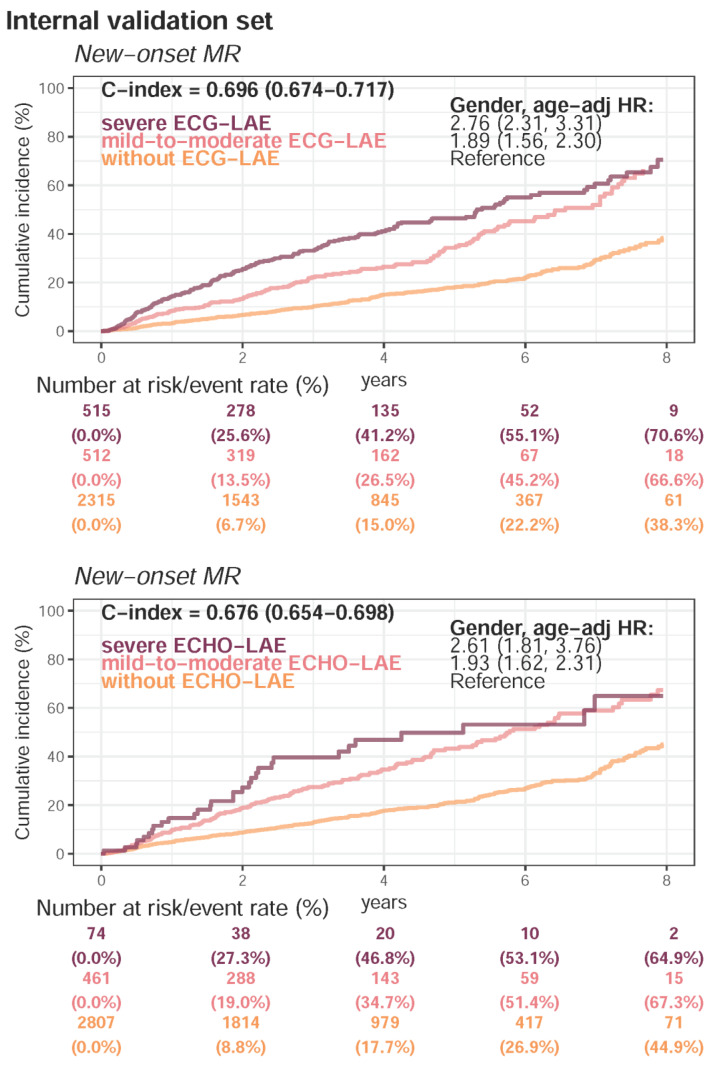
Kaplan–Meier curves for each severity of electrocardiogram-based left atrium enlargement (ECG-LAE) and echocardiography-based left atrium enlargement (ECHO-LAE) on new-onset mitral regurgitation (MR) in internal validation set. The cutoff points of without, mild-to-moderate, and severe LAE were defined as 45 and 55 mm, respectively. The C-index was calculated based on the continuous value combined with sex and age. The table shows the at-risk population and cumulative risk for the given time intervals in each risk stratification.

**Figure A7:**
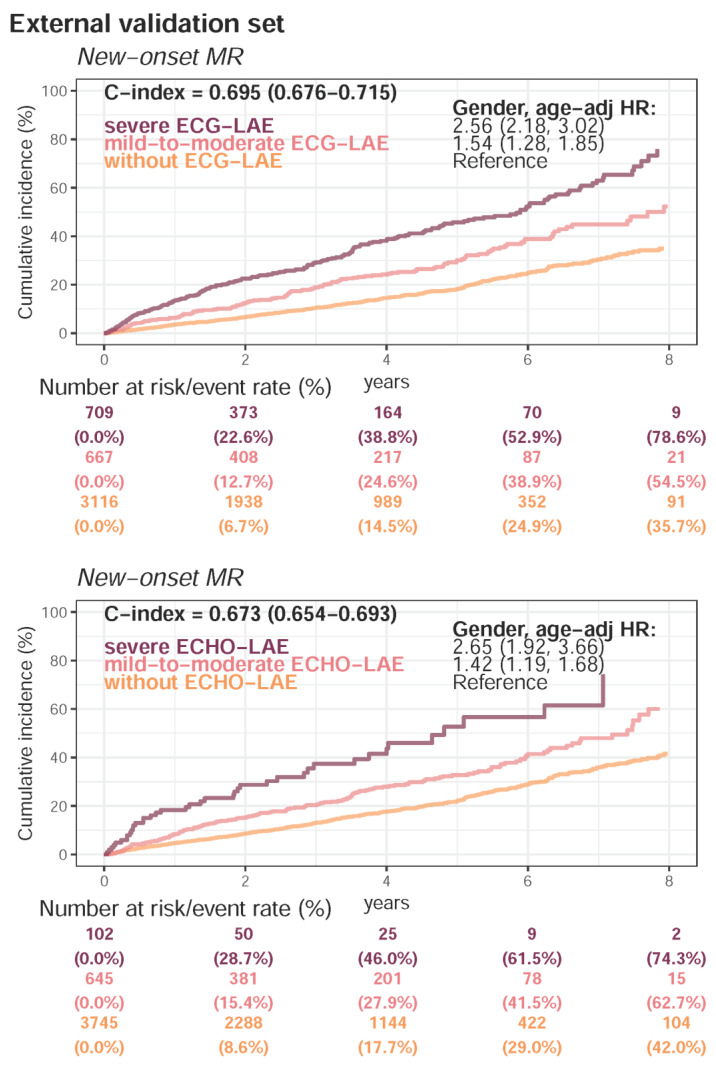
Kaplan–Meier curves for each severity of electrocardiogram-based left atrium enlargement (ECG-LAE) and echocardiography-based left atrium enlargement (ECHO-LAE) on new-onset mitral regurgitation (MR) in external validation set. The cutoff points of without, mild-to-moderate, and severe LAE were defined as 45 and 55 mm, respectively. The C-index was calculated based on the continuous value combined with sex and age. The table shows the at-risk population and cumulative risk for the given time intervals in each risk stratification.

**Figure A8:**
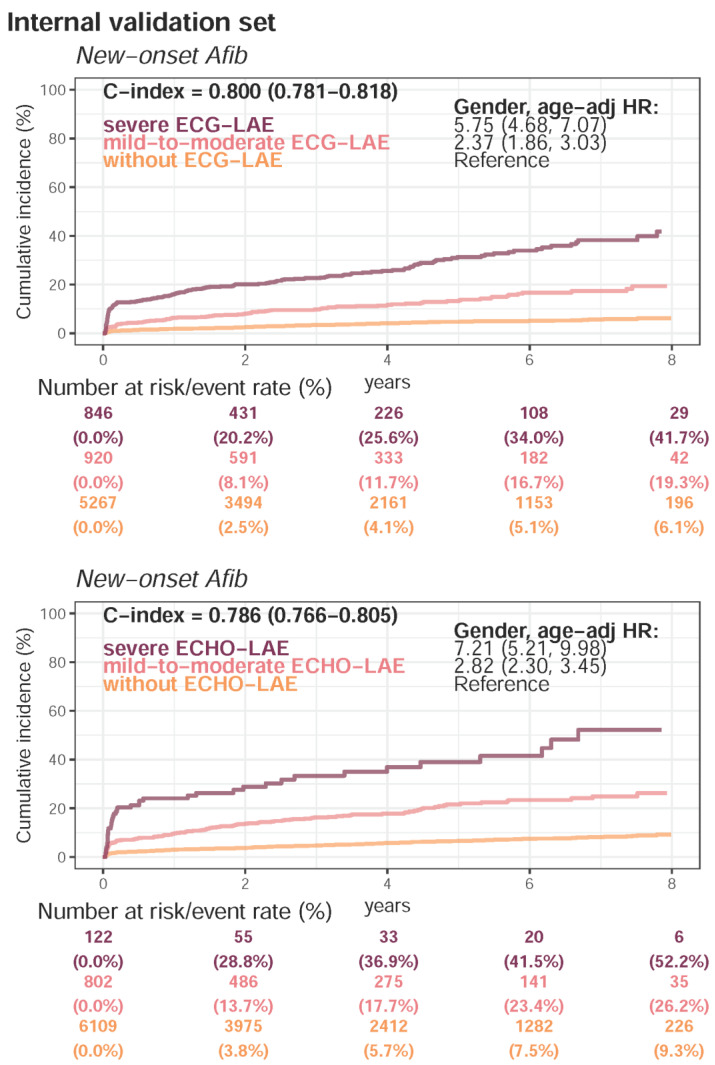
Kaplan–Meier curves for each severity of electrocardiogram-based left atrium enlargement (ECG-LAE) and echocardiography-based left atrium enlargement (ECHO-LAE) on new-onset atrial fibrillation (Afib) in internal validation set. The cutoff points of without, mild-to-moderate, and severe LAE were defined as 45 and 55 mm, respectively. The C-index was calculated based on the continuous value combined with sex and age. The table shows the at-risk population and cumulative risk for the given time intervals in each risk stratification.

**Figure A9:**
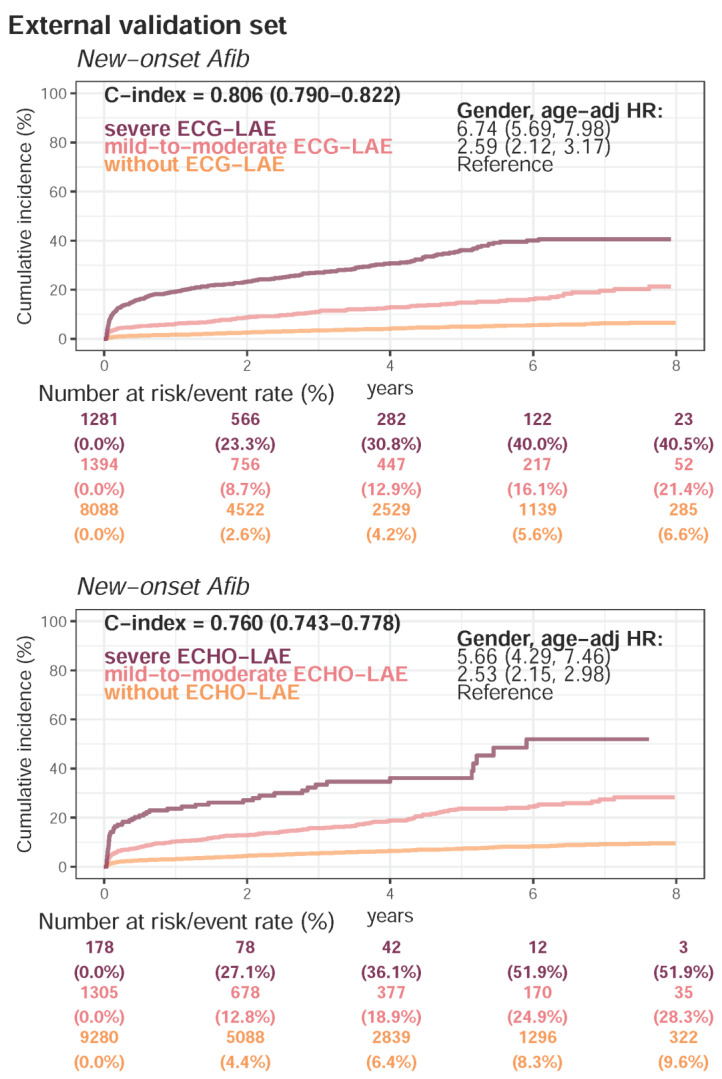
Kaplan–Meier curves for each severity of electrocardiogram-based left atrium enlargement (ECG-LAE) and echocardiography-based left atrium enlargement (ECHO-LAE) on new-onset atrial fibrillation (Afib) in external validation set. The cutoff points of without, mild-to-moderate, and severe LAE were defined as 45 and 55 mm, respectively. The C-index was calculated based on the continuous value combined with sex and age. The table shows the at-risk population and cumulative risk for the given time intervals in each risk stratification.

**Figure A10:**
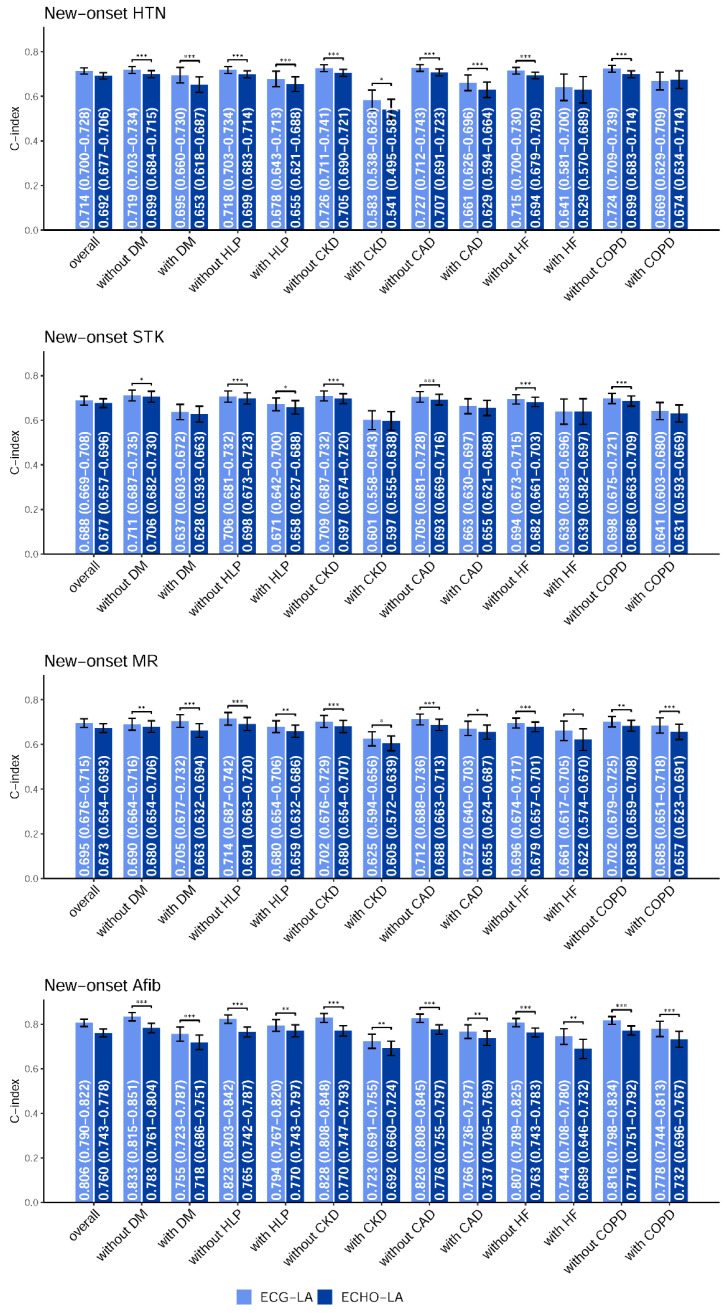
Stratified analysis for the C-index comparison between electrocardiogram-based left atrium (ECG-LA) diameter and echocardiography-based left atrium (ECHO-LA) diameter on new-onset complications in external validation set. The analyses were stratified by the disease histories of the populations. The C-index was calculated based on the ECG-LA/ECHO-LA combined with sex and age. *: *p* < 0.05; **: *p* < 0.01; ***: *p* < 0.001. The overall population analyses were performed with an unstratified Cox proportional-hazards model.

## Data Availability

The data in this study cannot be shared publicly due to the privacy of patients who participated in the study.

## Appendix B. Deep Learning Model Implementation

Our DLM is a 1D-convolution neuron network with dense connections between neuron layers, which had ECG lead blocks designed to extract the morphology features from an ECG. Our DLM consisted of 12 ECG lead blocks which shared weights among those lead blocks as ECG12Net [21]. We extended the ECG lead block with the specific nonshared neuron layers for improving the features extracting ability. The final temporal features of each ECG lead blocks are integrated with global average pooling. After integration, the final feature maps of 12 ECG leads are weighted summed with the attention blocks as ECG12Net [21]. The attention blocks had a hierarchical attention module to weight the final feature maps from ECG lead blocks, which had a fully connected layer with eight neurons, a batch normalization layer, and a ReLU layer. A fully connected layer with one neuron was used after a ReLU layer to generate the attention scores of each ECG lead. Finally, the final feature map from ECG lead blocks was weighted summed by simple multiplication of the attention scores of each ECG lead. Moreover, a dropout layer was added for each lead before weighted sum to prevent the model only using few parts of the ECG lead to predict outcome. The dropout rate was set as 0.5. The weighted sum is followed by a fully-connected layer with sigmoid functions.

## Appendix C. Categorywise Encoding Technology

We used categorywise encoding technology to encode the LA diameter. The continuous variable of LA was converted into ordinal variables with 20 categories. The DLM was designed to output the probability of each category. The last level was the maximal of the defined range, and the first level was the minimum of the defined range. The defined range of LA is from 20 to 65 mm, and the interval of each level was 2.25 mm. For example, a LA of less than 20 mm is coded as [0, 0, 0, 0, 0, 0, 0, 0, 0, 0, 0, 0, 0, 0, 0, 0, 0, 0, 0, 0], a LA of greater than 65 mm is coded as [1, 1, 1, 1, 1, 1, 1, 1, 1, 1, 1, 1, 1, 1, 1, 1, 1, 1, 1, 1], a case with a LA of 30 mm will be encoded as [1, 1, 1, 1, 1, 0, 0, 0, 0, 0, 0, 0, 0, 0, 0, 0, 0, 0, 0, 0], and so on. A fully-connected layer with 20 neurons followed by sigmoid function was used after the weighted sum, and we used the cross-entropy as the loss function. The final prediction value is the sum of sigmoid outputs multiplied by 2.25 plus 20. For example, a prediction vector for LA is [1, 1, 1, 0.6, 0.2, 0, 0, 0, 0, 0, 0, 0, 0, 0, 0, 0, 0, 0, 0, 0], which corresponds to a LA of 28.55.
